# Tracing the Roots of Inquiry: A Historical Review of Nursing Research Through Professionalisation Theory

**DOI:** 10.1111/nin.70160

**Published:** 2026-08-30

**Authors:** Raghad Almushawah, Dilla Davis, Ehsan Khan

**Affiliations:** ^1^ Department of Adult Nursing, Florence Nightingale Faculty of Nursing, Midwifery and Palliative Care King's College London London UK; ^2^ Department of Nursing, College of Applied Medical Sciences University of Bisha Bisha Saudi Arabia

**Keywords:** artificial intelligence, COVID‐19, evidence‐based practice, nursing history, nursing profession, nursing research, professionalisation theory

## Abstract

Nursing research has developed unevenly across history, shaped by shifting institutional structures, professional aspirations and societal demands. Yet existing historical reviews have largely adopted descriptive approaches, while few have applied an explicit theoretical lens to explain why this unevenness persists. This paper addresses that gap by examining the historical development of nursing research through Greenwood's trait‐based professionalisation framework. Drawing on a narrative historical review spanning seven chronological periods, the analysis reveals a recurring pattern of *partial professionalisation*; nursing has consistently generated specialised knowledge yet has struggled to secure organisational infrastructure to embed and control that knowledge within clinical practice. The historical analysis suggests that knowledge production alone within a profession does not guarantee epistemic control.

## 1 Introduction

Nursing, as both a profession and a discipline, occupies a paradoxical position within modern healthcare. It is built on specialised knowledge and clinical expertise, yet it struggles to define the terms of its own epistemic authority. While clinical practice has often taken centre stage in the public understandings of nursing, the development of nursing research tells a parallel story, one of intellectual labour, evolving identity and an ongoing negotiation of knowledge and authority within the wider academic and healthcare communities. Nursing has consistently produced specialised knowledge while struggling to secure the organisational and cultural conditions needed to control how that knowledge is embedded and legitimised in practice, a recurring epistemic asymmetry this paper conceptualises as partial professionalisation.

Caring for the sick long predates the emergence of nursing as a formal profession; ancient societies maintained organised care systems grounded in their own systematic knowledge long before professional structures existed (Donahue 2011; Baly 1995). Historical traditions point to early instances of structured caregiving, such as Rufaida Al‐Aslamia in early Islamic history (Jan 1996), Sushruta's trained attendants (Singh 2017) and Phoebe in early Christian texts (Osiek 2006). Historical analyses of ancient Egyptian nursing and midwifery also reveal clinically oriented knowledge bases, including documented training systems and professional hierarchies (Abdul et al. 2015; Elhabashy and Abdelgawad 2019).

These caregiving traditions were underpinned by their own bodies of knowledge and expertise, although they were not recognised as formal nursing knowledge within the frameworks that later came to dominate the professionalisation of nursing. What changed over time was not the presence of knowledge but the form that knowledge took and the institutional structures through which it was recognised and legitimised.

### 1.1 Purpose of the Review

Despite important turning points in the development of nursing as a profession, nursing research did not develop evenly over time, particularly in terms of institutional support, integration into practice and influence on professional authority (Song et al. 2025; WHO 2020; McSherry and Douglas 2011; Estabrooks 1999; Rafferty 1996). Existing historical reviews have largely addressed this unevenness descriptively, yet an explicit theoretical lens to explain why it persists or what it reveals about nursing's professional authority remains absent. Without such a lens, the recurring pattern of knowledge production without epistemic control that has shaped nursing's development remains underexamined.

This paper therefore addresses that gap by examining the historical development of nursing research through Greenwood's (1957) trait‐based professionalisation framework, chosen for its focus on knowledge development as the foundation of professional legitimacy, to conceptualise the recurring epistemic asymmetry between knowledge production and epistemic control as partial professionalisation.

### 1.2 Theoretical Framework: Greenwood's Trait‐Based Framework

The formal sociological study of professions emerged in the 1930s with trait‐based approaches that sought to identify the essential characteristics distinguishing professions from other occupations (Carr‐Saunders and Wilson 1933). Although these approaches shaped early thinking on professions, they were later criticised for their reliance on subjective criteria to certify occupational status (Abbott 1988; Larson 1977; Hall 1968).

Through the mid‐20th century, functionalist perspectives shifted attention to the profession's role in maintaining social order and commitment to altruistic service (Parsons 1939). Central to this view was the argument that the privileged status afforded to professions is justified by the trust society places in the professional's knowledge and judgement (Freidson 1970). Subsequent theorists argued that functionalist accounts present professional status as stable and consensual, thereby overlooking power relations and historical contingencies through which professional authority is constructed (Freidson 2001; Macdonald 1995; Larson 1977).

In response to these critiques, Abbott's (1988) jurisdictional theory marked a significant shift in the sociology of professions, centring professional identity not on a fixed set of traits but on a profession's capacity to secure and maintain control over specific domains of work. This positions the development of professions as inherently relational within a shared system, in which the work claimed by one profession directly affects the boundaries of another (Abbott 1988). While Abbott's (1988) jurisdictional theory is valuable for examining how professions compete for control over domains of work, its focus on inter‐professional competition is not the question this review asks: the concern here is not how nursing has competed for jurisdiction against other occupations but how its knowledge base has developed historically relative to its own claims to authority.

For that purpose, this review returns to Greenwood's (1957) trait‐based framework, not as a checklist for certifying professional status but as an analytic lens for tracing professionalisation as a developmental process in relation to epistemic growth. It recognises that while many occupations pursue professional recognition, relatively few succeed in securing authority and legitimacy (Wilensky 1964; Greenwood 1957). Greenwood identified five attributes through which occupations move towards professional legitimacy: a systematic body of theory, professional authority, community sanction, ethical regulation and professional culture.

Of these, particular attention is given to the argument that professional practice is sustained not by experience alone, but by an internally coherent system of knowledge from which professional skill and judgement ultimately derive, a process he termed ‘body of theory’ (Greenwood 1957, 46). This argument is especially relevant to nursing, where the development of a research‐based knowledge base has not consistently translated into equivalent professional authority or institutional recognition, the asymmetry this review terms partial professionalisation.

## 2 Methods

A narrative historical review was conducted to explore the evolution of nursing research through the lens of Professionalisation Theory, guided by Greenwood's trait‐based framework. Relevant literature was identified through searches of PubMed, Scopus and Web of Science. In addition, archival materials from organisations such as the World Health Organization (WHO) and the American Nurses Association (ANA) were reviewed to identify further relevant sources.

Selection was guided by conceptual relevance and historical significance rather than by exhaustive coverage, consistent with the aims of a narrative historical review (Grant and Booth 2009). Identified concepts were progressively refined and grouped into broader themes through repeated comparison across sources and historical periods. Themes were then organised chronologically and interpreted through a trait‐based professionalisation framework.

## 3 Results

**Figure 1:**
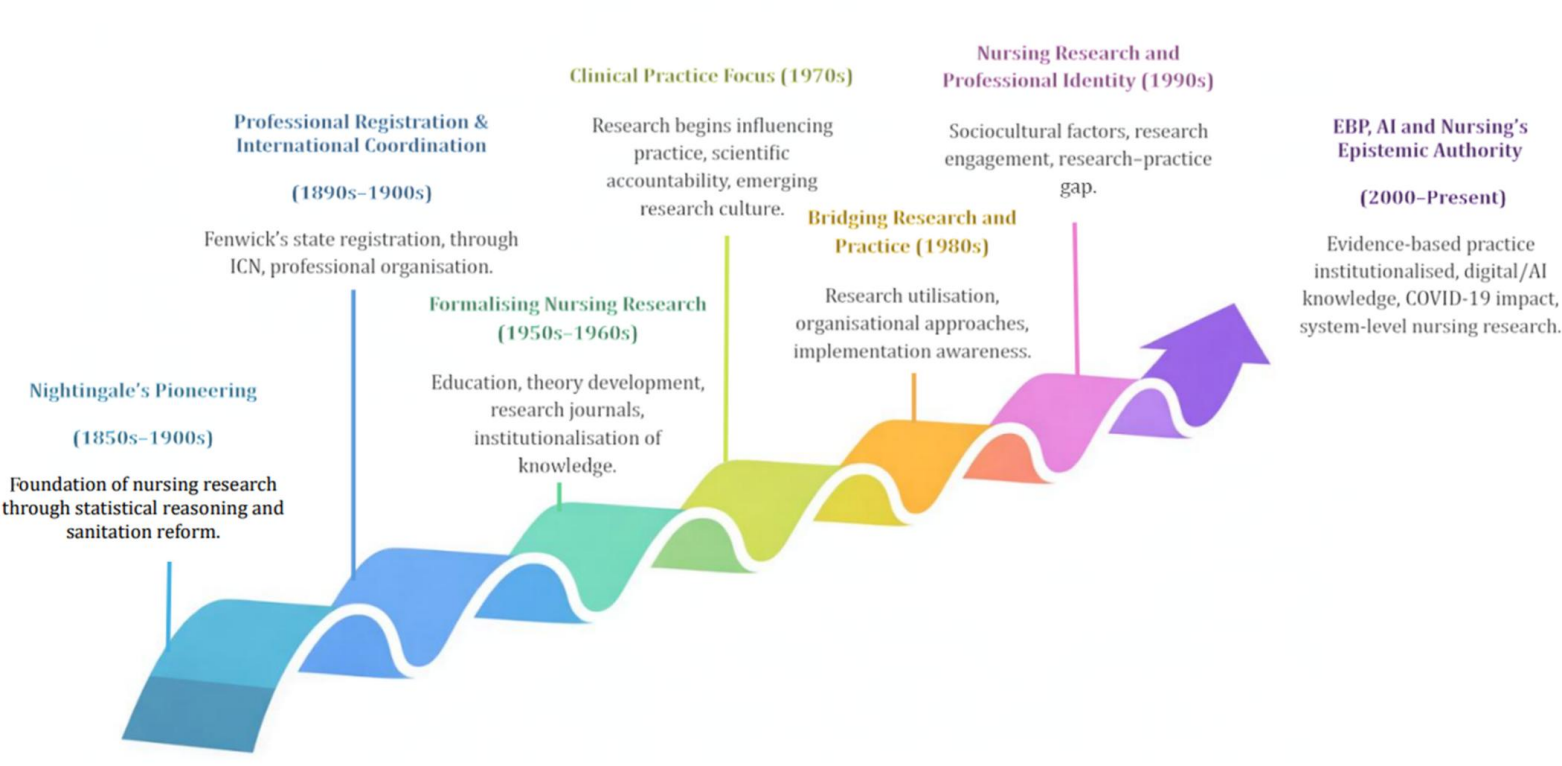
Timeline of key periods in the historical development of nursing research (1850s–present). (The graphic template was AI‐generated [ChatGPT]; the timeline content and text are written by the authors based on the analysis of each historical period.)

The review of historical sources traces how nursing research developed alongside the profession's evolving claims to legitimacy. Over time, nursing's efforts to understand and improve its own practice gradually shaped a distinct professional identity grounded in knowledge, inquiry and accountability. Viewed through the lens of Greenwood's (1957) trait‐based theory, seven periods emerged (Figure 1), each representing a shift in how nursing knowledge was produced, legitimised or contested. These stages demonstrate how changes in society and healthcare institutions influenced what counted as nursing knowledge and who was seen as responsible for producing it. The following section traces this progression chronologically through seven periods, highlighting the main developments and turning points within each era.

### 3.1 Florence Nightingale: Statistics, Reform and Professional Knowledge

Florence Nightingale is an important figure in nursing research as she was among the first to use statistical and quantitative methods to improve healthcare practices. This approach in her time was essential for establishing authority within scientific and political communities. During the Crimean War, she combined data, political access and a commitment to reform in ways that began to reframe nursing as a practice capable of generating knowledge that speaks the language of science and policy.

During the Crimean War, the British and French forces were deployed to support Turkey against Russia (Ellis 2020; McDonald 2019). Later that year, reports highlighted the severe conditions that British soldiers endured, in contrast to the superior medical care provided to French troops (Ellis 2020). The military hospitals lacked basic medical supplies, were unsanitary and overcrowded. Widespread infectious diseases including typhoid, dysentery and cholera increased fatality rates more than battlefield casualties (Kent‐Wilkinson 2021; Brasseur 2005).

Nightingale highlighted poor sanitation as a leading cause of soldier mortality and implemented handwashing and strict hygiene protocols to improve conditions in British Army Hospitals (Martini and Lippi 2021; Monteverde and Gallagher 2020). Bostridge (2008) documents that awareness of unsanitary conditions in military hospitals was already present before Nightingale's arrival. What distinguished Nightingale's contribution was her access to politicians in London and the resources to implement reforms at a scale and speed that others could not (Bostridge 2008). Mortality rates reportedly dropped from 42.7% to 2.7% within a few months (Ellis 2020; Martini and Lippi 2021).

Nightingale's primary renown among her contemporaries was as a statistician, as statistical science was the dominant form of empirical inquiry in 19th‐century Western Europe (Bostridge 2008; Palmer 1977; Kopf 1916). Her iconic polar area graph, originally titled *Diagram of the Causes of Mortality in the Army in the East* (Martineau and Nightingale 1859), revolutionised the use of statistical graphics in public health advocacy (Brasseur 2005). The statistical graph presented data on soldiers' monthly mortality rates using wedges and colour‐coding, which made the message clear and accessible, enabling even non‐statisticians to recognise the dominant impact of poor sanitation on soldier mortality (Brasseur 2005).

Following the war, Nightingale continued this work through a detailed analysis of hospital mortality, resulting in her treatise, *Notes on Matters Affecting the Health, Efficiency and Hospital Administration of the British Army* (McDonald 2015; Brasseur 2005). Nightingale's systematic approach to data collection and analysis has been recognised by nursing scholars as anticipating the principles that would later underpin evidence‐based practice (Mackey and Bassendowski 2017; Woodham‐Smith 1951).

Viewed through Greenwood's (1957) framework, Nightingale's contribution marks a significant moment in which systematic knowledge began to function as a source of professional authority. Her statistical work demonstrated that nursing could produce the kind of evidence that institutions recognised as legitimate, thereby satisfying Greenwood's (1957) foundational trait of a systematic body of theory.

However, this period also revealed an important limitation. Nightingale's authority was derived from her individual social position and political connections rather than from any institutional infrastructure capable of sustaining or extending that knowledge. This tension marks what this paper conceptualises as an early instance of *partial professionalisation*: the knowledge had begun to emerge, but the organisational conditions required to control it within a recognised professional framework was underdeveloped.

### 3.2 Professional Registration and International Coordination: Ethel Fenwick and the International Council of Nurses (1890s–1900s)

During the late 19th century, Ethel Gordon Fenwick became one of the key figures in shaping what would come to be recognised as the modern professional identity of nursing. She championed state registration, standardised education and international collaboration. Through these efforts, nursing moved beyond its roots as a philanthropic pursuit towards becoming a self‐regulating profession. As a former matron of St Bartholomew's Hospital, Fenwick founded the British Nurses' Association in 1887, advocating for formal education, licensure and public recognition of nurses as skilled professionals rather than domestic helpers (Rafferty 1996; Maggs 1987).

Her persistence over more than three decades ultimately led to the Nurses Registration Act of 1919, which created the first statutory register of trained nurses in England and Wales (Baly 1995). It is worth noting, however, that Fenwick's path to registration was complicated in part by Nightingale herself, who was firmly against state registration; she maintained that a register could not capture the moral and character qualities she believed essential to nursing (Bostridge 2008; Rafferty 1996). Nevertheless, this landmark achievement firmly established nursing within the framework of professional regulation (Baly 1995).

Speaking at the Matrons' Council Conference in London in 1899, Fenwick proposed the establishment of the International Council of Nurses (ICN) to unite nurses globally and to institutionalise control over professional standards. She argued that nurses' care for the sick depended on their competence, trustworthiness and ability to perform their duties to the highest benefit of those in their care and to achieve these goals, two things were essential ‘there should be recognised systems of nursing education and of control over the nursing profession’ (Fenwick 1901, 788). Her call for recognised systems of education reframed nursing as an intellectual field, laying the groundwork for what Greenwood (1957) described as a systematic body of knowledge, framing education not merely as training but as the mechanism through which nursing could claim epistemic authority.

The ICN was founded in 1899 as the first international organisation for healthcare professionals, with the United Kingdom, the United States of America and Germany as its founding members (International Council of Nurses 2023). In outlining the objectives of the newly established ICN, Fenwick as its president identified two central purposes: to facilitate communication and cooperation among nurses of all nations and ‘to provide opportunities for nurses to meet together from all parts of the world to confer upon questions relating to the welfare of their patients and their profession’ (Fenwick 1901, 789).

These aims reflected her conviction that professional progress was inseparable from collective deliberation and mutual accountability. As she later emphasised, Fenwick believed that both professions and nations could only thrive when individuals developed a shared sense of collective responsibility for their work and its standards (Fenwick 1901). For Fenwick, the ICN's first and most important task was ‘to confer upon questions relating to the definition of the basis of education and qualifications for a trained nurse’ (Fenwick 1901, 789).

Together, these developments show that Fenwick and her contemporaries were not only building professional structures but also shaping how nursing understood and presented itself as a discipline. Fenwick's work collectively addressed four of Greenwood's five traits, systematic knowledge through standardised education, authority through registration, social approval through international recognition and ethical regulation through professional governance. The remaining challenge lay in establishing a shared professional culture capable of sustaining and advancing these achievements beyond the influence of individual people.

### 3.3 The Mid‐20th Century: Federal Funding and the Institutionalisation of Nursing Research

The mid‐20th century marked a turning point in the institutionalisation of nursing research. What changed in this period was not the emergence of nursing research itself but the source of its support, shifting from philanthropic to federal funding (Gortner 1983). The first officially ratified ANA Code of Ethics in 1950, although devoid of explicit references to research, implicitly recognised the role of scholarly inquiry in professional practice. It emphasised the need for nurses to engage in continuous reading, study and investigation as essential components of their professional development (Smirnoff et al. 2007).

Growing concerns about the quality of care during this period highlighted the need for more systematic nursing education. This encouraged the development and advancement of an educational curriculum for nurses (Gortner 1983). Influential theorists such as Virginia Henderson and Hildegard Peplau further advanced the theoretical underpinnings of nursing practice (LoBiondo‐Wood and Haber 1998). Henderson articulated the functions of nursing beyond medical assistance, advocating for a clear definition of nursing's role in supporting individuals' health and independence (Henderson 1966). Peplau's work on interpersonal relations advanced the therapeutic nature of nurse–patient interactions (Peplau 1992). These advancements aligned with the launch of several nursing research journals and the formation of the ANA Commission on Nursing Research, which reflected the profession's growing dedication at the time to build a strong foundation for nursing research (LoBiondo‐Wood and Haber 1998).

Together, these developments represented a concerted effort to establish the theoretical and empirical foundations that Greenwood (1957) identifies as essential to professional legitimacy, the systematic body of knowledge from which professional skill and judgement derive. However, a significant challenge was the limited engagement of nurses with research literature. Reinkemeyer (1968) discussed that nurses as a professional group lacked a strong intellectual tradition. This absence of a well‐established research culture often resulted in ambivalence and at times, resistance towards research‐related developments (Reinkemeyer 1968). Stolley et al. (2000) later reflected on that period, arguing that these challenges might be due to the traditional view of nursing as a task‐oriented apprenticeship profession. As a result, theory was primarily used to support training rather than guide practice or research (Stolley et al. 2000).

The challenges of this period had deeper roots. Reverby (1987) documents that in the 1920s and 1930s, academic nursing leaders debated what nursing research should be, most notably at Columbia University, where Virginia Henderson argued that research questions should emerge from clinical practice. At the same time, Isabel Stewart believed students should capitalise on time‐and‐motion studies to determine the most efficient way to perform clinical procedures. Stewart's approach prevailed, orienting nursing research towards the standardisation of clinical procedures, a methodology that continued to shape nursing's epistemic identity well into the mid‐century (Reverby 1987). This uncertainty extended to the profession's research publications as Baer (1987) documents that the editors of Nursing Research journal debated whether the term nursing research referred to studies of nursing as an occupation or studies of nursing practice itself (Baer 1987).

Viewed through Greenwood's framework, this period marked an important step in nursing's professionalisation. Research infrastructure expanded, nursing theories emerged and federal funding strengthened the profession's knowledge base. However, ongoing uncertainty about the purpose of nursing research and limited engagement with research among practising nurses suggest that the growth of nursing knowledge was not always matched by a culture that fully supported its use. As a result, nursing made substantial progress towards professional legitimacy, yet elements of professionalisation remained incomplete.

### 3.4 The 1970s: Research Shifting Into Clinical Practice

Becker's (1971) perspective on professions emphasised that they were characterised by exclusive control over specialised, complex and difficult knowledge. This ‘monopoly’ of expertise distinguished professions from other occupations and justified their elevated status and societal role. In the 1970s, this idea raised questions about nursing's professional status, particularly when certain nursing tasks traditionally viewed as part of the profession were later taught to healthcare assistants on a competency‐only basis (Becker 1971; Lacey 1994). This shift implied that such tasks did not require the advanced theoretical knowledge or critical decision‐making typically associated with a professional role.

Central to professionalisation theory is the understanding that professions are sustained by a distinct and evolving body of knowledge that informs practice and underpins professional authority (Freidson 2001; Greenwood 1957). Consequently, the development and application of research became increasingly viewed as essential to strengthening nursing's professional foundation. Scholars such as Gortner (1974) and Abdellah and Levine (1979) argued that research should be central to professional practice, precisely because it was not yet embedded within everyday clinical work (Abdellah and Levine 1979; Gortner 1974).

Writing retrospectively, Closs and Cheater (1994) described nursing at the time as divided into two distinct subcultures, one for nurses and another for researchers, each with its own values and language. Despite efforts to bridge the gap, no clear definition of a nursing research culture in clinical practice was identified (Closs and Cheater 1994).

However, nursing research moved beyond theoretical discussions and began focusing on the practical application of findings in clinical settings. This transition was reinforced by the 1976 revision of the ANA Code of Ethics, which highlighted the responsibility of all nurses to contribute to the profession's expanding body of knowledge (American Nurses Association 1976; Jacobson et al. 2008). What was once viewed as a primarily academic pursuit gradually gained recognition as a justified practice in healthcare (Chance and Hinshaw 1980).

Furthermore, Gortner (1974) emphasised that nurses must be scientifically accountable at all levels of practice, which highlighted that safe, effective care requires more than just experience (Gortner 1974). In later years, scholars identified nursing research as the foundation for scientific accountability, providing the evidence base necessary to establish the scientific principles of nursing practice (Chenger 1988; Clark 1987; Goode et al. 1987; Hunt 1981).

Despite ongoing divisions between researchers and clinicians, the 1970s marked an important turning point in the relationship between nursing research and practice. Research was increasingly viewed as a means of improving patient care rather than solely as an academic activity, which, viewed through professionalisation theory, contributed to further developing the profession's systematic body of knowledge.

### 3.5 The 1980s: Bridging Research and Practice

The expanding focus on research became more evident in the late 20th century as growing healthcare costs and economic restrictions put greater pressure on nurses to rationalise the expenses related to nursing care. Nurses were expected to demonstrate the societal impact of their knowledge and skills to secure resources and maintain high standards of patient care (Chenger 1988). As a result, research became essential not only for improving clinical practice but also for validating nursing's contributions to broader healthcare outcomes.

Stolley et al. (2000) noted that it was not until the 1980s that nursing research began to focus significantly on patients and patient behaviour. This shift occurred as nurses recognised the complex relationship between behaviour, rehabilitation and recovery from illness. Historically, nursing practice had centred on identifying single causative agents when promoting health or preventing disease, despite an awareness of the multiple factors influencing health outcomes (Stolley et al. 2000).

In the United Kingdom, the predominant strategy for promoting research utilisation focused on educating individual nurses (Myco 1981). This approach was previously criticised by Caplan (1979), who referred to it as an ‘input–output’ model. Caplan argued that this model was inadequate because it overlooked broader systemic factors. He noted that bureaucratic, ethical, attitudinal and social considerations often took precedence over the actual value of the research being presented, limiting the model's effectiveness in practice (Caplan 1979).

Meanwhile, the Conduct and Utilization of Research in Nursing (CURN) study, led by Horsley (1983) in the United States, investigated research utilisation as an organisational, rather than an individual process. This study demonstrated the limitations of relying solely on individual nurses to integrate research into practice without sufficient institutional support (Horsley 1983). Rothman (1980) and Rogers (1983) similarly questioned the effectiveness of the laissez‐faire approach which left the responsibility for research utilisation entirely to individual practitioners. They argued that, for research to truly influence nursing practice, a more structured and organisation‐wide strategy was essential (Rogers 1983; Rothman 1980). A systematic body of nursing knowledge existed, but the institutional mechanisms needed to embed it in practice were absent, reflecting precisely the dynamic this paper conceptualises as partial professionalisation.

Stetler (1985) built on this viewpoint by highlighting that nursing research involved more than simply generating findings. She encouraged nurses to consider how research could be integrated into clinical practice through the broader concept of knowledge utilisation (Stetler 1985). Her work stressed the importance of applying research findings in ways that were relevant and actionable for nurses. Stetler's contribution was significant; by framing research utilisation as a professional responsibility rather than an individual choice, she implicitly addressed Greenwood's (1957) criterion of a shared professional culture, arguing that what distinguished a profession was not merely the possession of knowledge but the commitment to applying it.

The 1980s revealed a fundamental tension at the heart of nursing's professional journey as the profession had developed a substantial body of knowledge yet lacked the organisational infrastructure to translate that knowledge into consistent practice. Viewed through Greenwood's (1957) framework, this decade exposed the gap between possessing a systematic body of knowledge and securing the authority, social approval and shared professional culture needed to make that knowledge into professional authority.

### 3.6 The 1990s: Nursing Research and Professional Identity

In 1992, the WHO's report *Research Strategies for Health* drew attention to the growing pressure on healthcare professionals, including nurses, to actively engage in research (McSherry 1997). It cautioned that neglecting research could ultimately undermine health outcomes, wording this concern with an ‘alarming’ statement: ‘Neglect research and you neglect health’ (Webb and Mackenzie 1993, 8).

Bassett (1992) argued that nursing research needed to more closely reflect the realities of clinical practice, criticising the dominance of abstract and idealised theories within the literature that often fail to resonate with bedside nurses. When research appears disconnected from everyday practice, it risks being dismissed, regardless of its potential value. Bassett therefore emphasised that for research to have a meaningful impact, it must directly address the practical challenges nurses encounter in delivering care (Bassett 1992). This resonates with Greenwood's (1957) broader argument that a systematic body of knowledge functions as the foundation of professional authority only when it informs practice (Greenwood 1957). Therefore, knowledge that cannot reach those it is meant to guide cannot sustain the professional legitimacy it was built to support.

Hicks (1996) explored the sociocultural dynamics contributing to this issue, noting a perceived tension between the qualities associated with being a ‘good researcher’ and those attributed to being a ‘good clinician’. She suggested that this tension was partly rooted in traditional, gendered expectations within the predominantly female nursing workforce. Many nurses saw their primary responsibility as delivering direct patient care, with research viewed as a separate, specialised activity (Hicks 1996). This perception, coupled with demanding workloads, made it difficult for nurses to find time or motivation to engage in research.

From a professionalisation perspective, this divide had implications as Greenwood (1957) argued that professions are characterised not only by a specialised body of knowledge but also by a shared professional culture. When research and clinical practice are perceived as separate domains, the development of a unified professional identity becomes more difficult. The persistence of gendered assumptions surrounding research and care therefore reflected a broader cultural barrier to integrating research into nursing.

Clarke and Procter (1999) expanded on this issue by examining the relationship between research, practice development and clinical care. Their findings revealed a persistent gap between researchers and practitioners, with research often perceived as a time‐intensive, intellectually demanding task disconnected from the realities of clinical work. They argued that addressing this disconnect required stronger collaboration between researchers and frontline nurses, along with institutional support to integrate research into clinical practice. Only through these efforts, they concluded, could nursing fully realise the potential of research to enhance patient care and contribute to the profession's ongoing development (Clarke and Procter 1999).

Rather than representing isolated barriers, these debates reflect a deeper structural contradiction within nursing during the late 20th century. While the profession was being encouraged to define itself as knowledge‐driven and research‐informed, gendered expectations and clinical work structures continued to treat research as peripheral to ‘real’ nursing work. This tension revealed a gap between the profession's knowledge‐based aspirations and the structural conditions required to support them.

### 3.7 2000–Present: EBP, AI and Nursing's Epistemic Authority

Nursing research has changed since the turn of the millennium through three interconnected developments: the rise of EBP, the rise of artificial intelligence (AI) and the COVID‐19 pandemic. Together, these developments reshape where knowledge in nursing comes from and who holds authority over it, placing research as a central condition of nursing's legitimacy within the increasingly complex and technology‐driven healthcare systems.

Although the term ‘evidence‐based’ originated from medicine by a research group in the early 1990s (Beyea and Slattery 2013; Guyatt 1992), its translation into nursing required an epistemic reorientation. In response to concerns that early formulations of EBP privileged quantitative evidence and marginalised the theoretical and relational dimensions of nursing, Ingersoll (2000) proposed a revised definition tailored to the profession. She reframed evidence‐based nursing (EBN) as the ‘conscientious, explicit and judicious use of theory‐derived, research‐based information in clinical decision‐making’, taking into account patients' individual needs and preferences (Ingersoll 2000, 152).

McMenamin et al. (2019) portray the 2000s as a decade in which EBP shifted from an ideal to an institutional expectation. EBP became embedded at a systems level across healthcare institutions; in the United States, this was shaped by regulatory and accreditation bodies such as The Joint Commission, The Magnet Recognition Program and the Institute for Healthcare Improvement (McMenamin et al. 2019; Beyea and Slattery 2013). The ability to demonstrate the use of research in everyday practice became a defining professional demand (McMenamin et al. 2019).

Within Greenwood's (1957) trait‐based model, this shift strengthens two defining features of professional status: a coherent body of knowledge and recognised authority (Greenwood 1957). Therefore, by grounding practice in research‐derived evidence, nursing affirms its epistemic autonomy and reinforces its place within interprofessional hierarchies long dominated by medicine. Nursing research has increasingly turned towards producing knowledge that can be used directly in practice, including systematic reviews and the development of implementation science, repositioning the profession as a producer rather than a consumer of evidence (Melnyk and Fineout‐Overholt 2022; Polit and Beck 2020).

AI gained recognition in healthcare in the late 20th century and more recently in nursing research. It is broadly defined as computational systems that can perform tasks that normally require human intelligence (Nashwan et al. 2025). Initially incorporated into nursing through decision support systems and informatics, AI represents a new phase in the production and distribution of nursing knowledge (Ruksakulpiwat et al. 2025; Salvagno et al. 2023). It can shorten research cycles, expand analytical reach and enable anticipatory forms of inquiry, enabling collaboration between nursing and fields such as computer science, informatics and data science (Ahmed 2024). Large‐scale, system‐level and predictive forms of knowledge are becoming more prevalent in nursing inquiry, a shift from the field's traditional focus on small‐scale context‐bound studies (Kim et al. 2025; Dailah et al. 2024).

However, from a professionalisation perspective, the critical question is not what AI can do but who controls it. Ruksakulpiwat et al. (2025) note that nurses are often positioned as end‐users rather than co‐designers of AI systems, producing a persistent disconnect between this development and nursing experience (Ruksakulpiwat et al. 2025). This misalignment may risk relocating epistemic authority away from the profession itself. AI therefore represents both an opportunity to expand nursing's body of knowledge and a site of vulnerability; whether nursing will help shape these systems or, as has happened before, find itself implementing knowledge structures it had no part in designing remains an open question.

The COVID‐19 pandemic tested these transformations under conditions of extreme uncertainty. Beyond its clinical impact, the pandemic revealed major structural flaws in the way nursing research is incorporated into healthcare systems (Rosser et al. 2020). Clinical‐research nurses were redeployed into frontline roles, draining research expertise when nursing‐specific knowledge was urgently needed (Castro‐Sánchez et al. 2021). Early‐career researchers were also significantly affected, with studies interrupted, funding applications suspended and opportunities for international collaboration were limited (Castro‐Sánchez et al. 2021; Sun et al. 2017).

Simultaneously, the pandemic drove the need to advance technology in the healthcare sector. To maintain care continuity during lockdowns, nurses quickly adjusted to telemedicine platforms, remote monitoring and digital triage systems (Bashshur et al. 2020). These changes repositioned nurses as facilitators of technology‐enabled care and even expanded healthcare access to underserved communities. Additionally, COVID‐19 encouraged the incorporation of AI into standard practice, which helped with workflow efficiency, personalised interventions and predictive analytics (Ahmed 2023a, 2023b).

The situation highlighted a clear paradox: nurses were essential to delivering technology‐enabled care yet remained absent from producing research that shaped that care. Although nurses generated practice‐based insights during the pandemic, the profession lacked the structure needed to transform this knowledge on a wider scale (Rosser et al. 2020). This raises an important question: what actually constitutes research? Much of what nurses knew and shared during the pandemic did not take the form of formal studies at the time, yet it was grounded in clinical observation, experience and problem‐solving abilities. The informal networks through which nurses and physicians exchanged what was working and what was not can reasonably be understood as a form of practice‐based inquiry.

Castro‐Sánchez et al. (2021) describe the post‐pandemic period as a moment of reconstruction in which a different future for nursing research becomes possible (Castro‐Sánchez et al. 2021). These developments demonstrate how COVID‐19 fundamentally reshaped the profession's research agenda. Nursing research now reaches beyond bedside care to address systems design, workforce organisation and health equity. This shift strengthens nursing's role in negotiating professional authority within complex healthcare environments. In doing so, the pandemic links the historical evolution of nursing with the demands of the future, giving nurses new epistemic responsibilities and redefining the field as a producer of both clinical and societal knowledge.

Research is no longer viewed as an activity confined to academic settings but has become closely linked to how nurses evaluate and improve care. These developments have strengthened nursing's knowledge base and reinforced the profession's authority to make evidence‐informed decisions. At the same time, they have raised new questions about how knowledge is generated, who controls it and whose expertise is recognised within increasingly digital healthcare systems. As nursing continues to evolve, maintaining professional authority will depend not only on producing research but also on ensuring that nurses remain actively involved in shaping research agendas and knowledge systems that influence practice.

The reviewed sources were synthesised into seven major chronological periods reflecting the evolution of nursing research and its contribution to nursing's professionalisation. Table 1 presents a concise summary of each historical phase, its core focus, representative references and contributions to the development of nursing as a profession.

**Table 1:** Historical evolution of nursing research and its contribution to professionalisation.

	Historical period	Core focus	Contribution to professionalisation of nursing	References
1	Florence Nightingale: The pioneer of nursing research (1850–1900)	Pioneered the use of statistical methods to improve healthcare practice; demonstrated that nursing could generate knowledge recognised as legitimate by scientific and political institutions.	Established the epistemological foundation of nursing as a science‐based profession; demonstrated that systematic observation and evidence could define professional authority and social legitimacy.	Kent‐Wilkinson (2021), Martini and Lippi (2021), Ellis (2020), Monteverde and Gallagher (2020), McDonald (2015, 2019), Mackey and Bassendowski (2017), Bostridge (2008), Brasseur (2005), Palmer (1977), Woodham‐Smith (1951), Kopf (1916) and Martineau and Nightingale (1859)
2	Professional registration and international coordination: Ethel Gordon Fenwick and the ICN (1890s–1900s)	Advocacy for state registration, standardised education and global cooperation; establishment of the ICN as the first international professional body for nurses.	Transformed nursing from a philanthropic pursuit into a self‐regulating profession; institutionalised education, ethics and collective authority. Principles later central to professionalisation theory.	International Council of Nurses (2023), Bostridge (2008), Rafferty (1996), Baly (1995), Maggs (1987) and Fenwick (1901)
3	The mid‐20th century: Formalising nursing research (1950s–1960s)	Development of nursing education, codes of ethics and theory‐based frameworks; formation of nursing research commissions and journals.	Formalised nursing as a knowledge‐based profession; emphasised education, reflection and ethics as foundations of professional autonomy. Though persistent uncertainty about the purpose and ownership of nursing research meant professionalisation remained incomplete.	Smirnoff et al. (2007), Stolley et al. (2000), LoBiondo‐Wood and Haber (1998), Peplau (1992), Baer (1987), Reverby (1987), Gortner (1983), Reinkemeyer (1968) and Henderson (1966)
4	The 1970s: Shifting research into clinical practice	Introduction of structured research programmes and growing awareness of research utilisation in practice; differentiation between academic and clinical nursing cultures.	Signalled the shift from theory‐based nursing to practice‐integrated research; strengthened accountability and redefined nurses as scientific contributors.	Jacobson et al. (2008), Freidson (2001), Lacey (1994), Closs and Cheater (1994), Chenger (1988), Clark (1987), Goode et al. (1987), Hunt (1981), Chance and Hinshaw (1980), Abdellah and Levine (1979), American Nurses Association (1976), Gortner (1974) and Becker (1971)
5	The 1980s: Bridging research and practice	Movement towards systematic research utilisation; organisational frameworks (CURN project); diffusion of innovation models.	Institutionalised research translation; established nursing research as both individual and organisational responsibility; promoted scientific accountability.	Stolley et al. (2000), Chenger (1988), Stetler (1985), Horsley (1983), Rogers (1983), Myco (1981), Rothman (1980) and Caplan (1979)
6	The 1990s: Nursing research and professional identity	Cultural and gendered dimensions of research engagement; barriers to integrating research into practice.	Framed research as an element of professional identity; highlighted sociocultural barriers, workload and institutional factors affecting engagement.	Stolley et al. (2000), Clarke and Procter (1999), McSherry (1997), Hicks (1996), Webb and Mackenzie (1993) and Bassett (1992)
7	2000–Present: EBP, AI and Nursing's Epistemic Authority	Institutionalisation of EBP; emergence of AI in nursing research; impact of COVID‐19 on nursing's research infrastructure and epistemic authority.	Research became a condition of professional legitimacy; EBP strengthened nursing's knowledge base while AI and COVID‐19 exposed persistent vulnerabilities in the profession's capacity to control its own knowledge agenda.	Kim et al. (2025), Nashwan et al. (2025), Ruksakulpiwat et al. (2025), Ahmed (2023a, 2023b, 2024), Dailah et al. (2024), Salvagno et al. (2023), Melnyk and Fineout‐Overholt (2022), Castro‐Sánchez et al. (2021), Rosser et al. (2020), Polit and Beck (2020), Taylor et al. (2020), Turale et al. (2020), McMenamin et al. (2019), Sun et al. (2017), Fineout‐Overholt et al. (2005), Ingersoll (2000), Melnyk et al. (2000) and Guyatt (1992)

## 4 Discussion

This review reinterprets the historical development of nursing research as a professional project through which nursing has sought to establish epistemic authority through its systematic body of knowledge. From Nightingale's statistical work to Fenwick's insistence on education and regulation, research has persisted as central to nursing's claim to professional legitimacy. Yet, as the subsequent decades reveal, the institutionalisation of research has remained uneven. Periods of intellectual expansion have repeatedly coexisted with institutionalised marginalisation, particularly in settings where clinical demands overshadow knowledge production. This paper conceptualises this recurring pattern as *partial professionalisation*: the systematic production of knowledge without consistent control over how that knowledge is embedded and legitimised in practice.

Across the seven periods examined, a consistent structural pattern emerges: nursing generated knowledge but lacked the organisational conditions to fully control how that knowledge was defined, applied and legitimised. This pattern did not resolve with time—it restated itself in new forms. EBP institutionalised the expectation that nursing knowledge must be evidence‐based, yet the framework nursing adopted to prove epistemic legitimacy was borrowed from medicine rather than generated from within the profession itself. AI is now reshaping who produces nursing knowledge and how, but nurses are frequently positioned as end‐users. COVID‐19 then exposed what happens when the infrastructure supporting nursing knowledge is under pressure, the profession became invisible epistemically at precisely the moment it was most visible clinically. Each development restates the same underlying tension: nursing generates knowledge but does not consistently control the conditions under which that knowledge is legitimised.

These concerns reflect something deeper than practical challenges. Berthelsen and Hølge‐Hazelton (2017) highlight the complexity of defining culture, emphasising that adopting a cultural perspective helps to explain why certain phenomena occur within specific contexts (Berthelsen and Hølge‐Hazelton 2017). Nursing culture not only reflects shared assumptions about care but also shapes what is considered legitimate knowledge and who is seen as a ‘research‐minded’ practitioner. The barriers to nursing's epistemic authority are therefore not only structural but cultural; nurses are consistently positioned as users of knowledge rather than producers.

Although nursing has developed a distinctive research base, the persistent gap between research and clinical practice shows that producing knowledge alone is not enough to secure epistemic control. Across different periods, nursing generated theory, evidence and methodological expertise, yet struggled to embed this knowledge within the everyday structures of care (Renolen et al. 2018; McSherry and Douglas 2011; Doane and Varcoe 2008). Research was often created in academic or specialist spaces, while clinical work remained shaped by service demands, time pressures and managerial priorities.

This pattern of partial professionalisation is not incidental to nursing's history; nursing possessed a growing body of specialised knowledge, but it did not consistently control how that knowledge is used in practice. Rather than fully monopolising epistemic authority, nursing has often seen this authority shaped through its interactions with the wider systems that increasingly influence how knowledge is produced and used. Nursing's historical trajectory therefore challenges the idea that knowledge possession alone is sufficient for professional authority, as authority depends not merely on expertise but on jurisdiction over its generation and mobilisation (Greenwood 1957).

## 5 Implications in Clinical Practice

The pattern this review identifies across seven historical periods suggests that nursing's challenge has not been a lack of knowledge production but a difficulty in securing the organisational and cultural conditions through which that knowledge becomes professionally authoritative.

The critique of the input–output model in the 1980s and the redeployment of research nurses during COVID‐19 reflect the same underlying issue separated by four decades. When research engagement depends on individual motivation rather than institutional structure, knowledge production tends to remain disconnected from practice. This suggests that protected research time and embedded clinical‐research roles are likely to matter more than individual motivation alone.

The cultural dimension appears equally persistent. The gendered framing this review traces from the 1990s onward, in which care is positioned as vocation and research as someone else's domain, has shaped engagement with research in ways that structural reform alone cannot fully address. A professional culture that positions nurses as producers of knowledge rather than consumers appears to require a fundamental shift and sustained investment in how nurses are educated, supported and recognised as producers of knowledge.

The emergence of AI raises a version of the same question this review has traced throughout. The historical record suggests a risk that nursing could once again find itself implementing a knowledge system it had little part in designing, unless the profession secures a more active role in shaping how that system is built. The capacity to shape the systems through which nursing knowledge is produced and legitimised is precisely what this review has shown to be persistently absent across nursing's professional history.

## 6 Conclusion

From Nightingale's polar area graphs to the algorithmic systems now reshaping clinical decision‐making, the profession has consistently found itself at the edge of epistemic authority, close enough to demonstrate knowledge but not close enough to control it. This is what this paper has termed partial professionalisation: not a failure of ambition or effort but a persistent gap between what nursing knows and what nursing is permitted to claim. The informal knowledge networks that emerged during COVID‐19, where nurses shared what worked across message boards and communication channels often ahead of formal publication, offer perhaps the most recent example of this gap.

Greenwood (1957) argued that a systematic body of knowledge is the foundation of professional legitimacy. What this review suggests is that, for nursing at least, this foundation, on its own, has not been sufficient; the institutional and cultural conditions needed to sustain it have remained persistently underdeveloped. Until nursing controls the conditions under which its knowledge is legitimised, the paradox this paper opened with is likely to remain unresolved.

## Funding

The authors have nothing to report.

## Ethics Statement

Ethical approval and informed consent were not required for this study, as it is based on a review and interpretation of published literature and archival sources.

## Conflicts of Interest

The authors declare no conflicts of interest.

## Disclosure of Artificial Intelligence (AI)

The authors used AI tools (ChatGPT and Claude) to assist with language clarity in parts of the manuscript and to generate the decorative graphic template used in Figure 1. All intellectual content, synthesis, interpretation and analytical framing are the authors' own. The authors reviewed all AI‐assisted content and the figure and take full responsibility for the final manuscript.

## Data Availability

Data sharing not applicable to this article as no datasets were generated or analysed during the current study.
